# A Waterborne Epoxy Composite Coating with Smart Corrosion Resistance Based on 2-Phenylbenzimidazole-5-sulfonic Acid/Layered Double Hydroxide Composite

**DOI:** 10.3390/molecules28135199

**Published:** 2023-07-04

**Authors:** Caiyou Ding, Jiongxin Wu, Yuan Liu, Xinxin Sheng, Xiaoling Cheng, Xiaoyan Xiong, Wenlin Zhao

**Affiliations:** 1 Guangdong Provincial Key Laboratory of Plant Resources Biorefinery, School of Chemical Engineering and Light Industry, Guangdong University of Technology, Guangzhou 510006, China; 2 Guangdong Provincial Key Laboratory of Functional Soft Condensed Matter, School of Materials and Energy, Guangdong University of Technology, Guangzhou 510006, China; 3 Center for Industrial Analysis and Testing, Guangdong Academy of Sciences, Guangzhou 510650, China; 4 GCH Technology Co., Ltd., Guangzhou 510540, China

**Keywords:** ZnAl-layered double hydroxide, 2-phenylbenzimidazole-5-sulfonic acid, anti-corrosion, water-based epoxy

## Abstract

In this study, ZnAl-layered double hydroxide (ZnAl-LDH) was functionalized with 2-phenylbenzimidazole-5-sulfonic acid (PBSA) to prepare ZnAl-PBSA-LDH using a simple one-step method. The electrochemical impedance spectroscopy (EIS) result of the solution phase demonstrated excellent corrosion inhibition performance of ZnAl-PBSA-LDH. Subsequently, 0.6 wt.% ZnAl-PBSA-LDH with shielding effects and active inhibition was incorporated into the water-based epoxy (WEP) for preparing the high-performance anti-corrosion coating (6-ZPL/WEP). The EIS test illustrated that the 6-ZPL/WEP coating maintained a high low-frequency impedance modulus (|Z_0.01 Hz_|) after 30 days of immersion, which is nearly two orders of magnitude higher compared to that of the blank coating. These results demonstrated that ZnAl-PBSA-LDH could efficiently improve the corrosion resistance of the WEP coating. Therefore, this study introduces new insights into the use of layered double hydroxides (LDHs) in the domain of anti-corrosion.

## 1. Introduction

Metal materials are widely used in diverse industrial fields, including parts support, oil pipelines, and ships. However, metal corrosion reduces the reliability of the metal, resulting in significant economic losses. Epoxy resin coatings have been widely employed as a preventive measure against metal corrosion due to their good adhesion, weather resistance, and chemical resistance [1]. However, traditional solvent-based epoxy resins release a volume of volatile organic compounds (VOCs) during the curing process, leading to pollution [2]. Therefore, environmentally friendly water-based epoxy (WEP) has gained attention as an anti-corrosion coating. Nevertheless, micropores and defects would be generated in the WEP coating during curing, leading to poor anti-corrosion performance. To address this issue, nanomaterials are introduced to enhance anti-corrosion performance [3,4,5,6,7].

Layered double hydroxides (LDHs) are a kind of layered substance made up of metal hydroxide layers with a positive charge and interlayer anions that fill the negative charges between the layers. LDHs have been widely utilized in the anti-corrosion field due to their wide surface expanse, adjustable surface chemistry, and effective ion exchange capacity [8,9,10,11,12,13,14]. Su et al. successfully inserted NO_2_^−^ into the interlayer of LDHs (LDH-NO_2_^−^) and introduced it into resin coatings. Electrochemical testing showed that the composite coating containing LDH-NO_2_^−^ exhibited excellent anticorrosion properties, resulting from the shielding properties and active inhibition of corrosion of LDH-NO_2_^−^ [15]. Buchheit et al. inserted decavanadate anions into LDH carriers and found that the VO_x_^−^ inhibitors significantly delayed the diffusion of chloride ions on the surface of aluminum alloys [16]. Results indicate that the LDH carriers could deliver the inhibitor effectively and sustain its release for improved long-term corrosion protection. These studies demonstrated the potential of LDHs loaded with corrosion inhibitors in corrosion protection applications. However, traditional corrosion inhibitors (phosphates, chromates, and dichromates) have significant impacts on the environment and human health [17]. As a result, there is a growing need to develop green and efficient corrosion inhibitors.

2-phenylbenzimidazole-5-sulfonic acid (PBSA) is applied to absorb UVB wavelengths in sunscreen due to its ability to absorb UVB, low toxicity, and relatively easy degradation [18,19,20]. PBSA possesses heteroatoms (N, S) that can combine with metal ions to form complexes to inhibit corrosion, enabling its application as a candidate for green corrosion inhibitors [18,21,22]. However, previous studies have not yet investigated the application of PBSA in the domain of corrosion protection.

Hence, ZnAl-PBSA-LDH was obtained by a single-step process, wherein ZnAl-LDH was simultaneously modified during the preparation of ZnAl-LDH using zinc chloride and aluminum chloride. The successful synthesis of ZnAl-PBSA-LDH was confirmed through chemical and physical characterization techniques. Additionally, EIS was employed to analyze the corrosion inhibition properties of ZnAl-PBSA-LDH in a 3.5 wt.% solution. Subsequently, ZnAl-PBSA-LDH was incorporated into WEP coatings to enhance their corrosion resistance. Lastly, the potential synergistic effect between PBSA and LDHs in improving the protective properties of the coatings was explored.

## 2. Results and Discussion

### 2.1. Characterization of MgAl-CO_3_^2−^-LDH, ZnAl-NO_3_^−^-LDH, and ZnAl-PBSA-LDH

**Figure 1:**
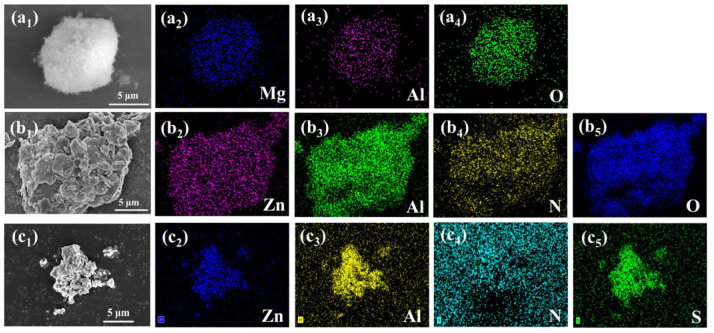
SEM images of (**a_1_**) MgAl-CO_3_^2−^-LDH and its EDS mapping corresponds to (**a_2_**) Mg, (**a_3_**) Al, and (**a_4_**) O, SEM images of (**b_1_**) ZnAl-NO_3_^−^-LDH and its EDS mapping corresponds to (**b_2_**) Zn, (**b_3_**) Al, (**b_4_**) N, and (**b_5_**) O. SEM images of (**c_1_**) ZnAl-PBSA-LDH and its EDS mapping corresponds to (**c_2_**) Zn, (**c_3_**) Al, (**c_4_**) N, and (**c_5_**) S.

The morphology of MgAl-CO_3_^2−^-LDH, ZnAl-NO_3_^−^-LDH, and ZnAl-PBSA-LDH was observed using SEM. Figure 1(b_1_) and Figure S1 show the microscopic morphology of ZnAl-NO_3_^−^-LDH, which resulted from the merging together of numerous small flake-like crystals [23]. After the insertion of PBSA, the morphology of the crystals was bulky (Figure 1(c_1_)). Furthermore, EDS mappings were utilized to analyze the elements distribution of the nanomaterial to confirm the success of modification. As Figure 1(c_2_–c_4_) shows, the presence of the Zn, Al, and S elements demonstrated the successful preparation of ZnAl-PBSA-LDH. The insertion of PBSA contributes to the improvement of the activity inhibition of LDHs.

**Figure 2:**
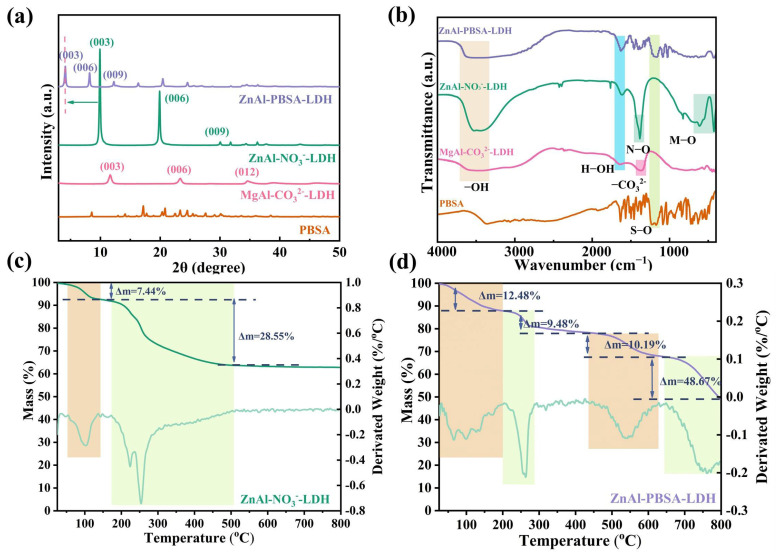
(**a**) XRD curves; (**b**) FTIR curves; TG-DTG curves of (**c**) ZnAl-NO_3_^−^-LDH and (**d**) ZnAl-PBSA-LDH.

The crystal structure of the nanomaterials was investigated using X-ray diffraction (XRD). As Figure 2a shows, diffraction peaks at (003), (006), and (012) were detected in the curve of MgAl-CO_3_^2−^-LDH, demonstrating the successful synthesis of MgAl-CO_3_^2−^-LDH. Furthermore, the characteristic peaks of ZnAl-NO_3_^−^-LDH were detected at 2θ values of 9.91°, 19.90°, and 30.04°, which were assigned to the (003), (006), and (009) peaks, respectively [24,25,26]. The (003) peak corresponds to a d-spacing value of 0.89 nm, consistent with previous reports [27,28,29,30]. After intercalation of PBSA, the (003) peak of ZnAl-PBSA-LDH shifted to 4.12°, indicating an increase in the d-spacing to 2.14 nm. This significant change indicates the successful intercalation of PBSA into the LDH layers.

Fourier-transform infrared spectroscopy (FTIR) was used to analyze the chemical composition of ZnAl-NO_3_^−^-LDH, ZnAl-PBSA-LDH, MgAl-CO_3_^2−^-LDH, and PBSA. In Figure 2b, the peaks observed at 3463 cm^−1^ and 1628 cm^−1^ in the FTIR curve of ZnAl-NO_3_^−^-LDH correspond to the stretching vibration of the −OH bond and the deformation vibration of water molecules, respectively [18,24,25,26,27,28,29,30,31,32]. Additionally, the N−O peak at 1387 cm^−1^ provided evidence for the successful intercalation of NO_3_^−^ ions into the LDH layers. Moreover, the stretching and bending modes of metal-oxygen (Zn−O and Al−O) are observed at a sequence of absorption bands that occur below 1000 cm^−1^ [33,34]. Collectively, these findings confirm the successful synthesis of ZnAl-NO_3_^−^-LDH. In comparison to ZnAl-NO_3_^−^-LDH, the FTIR spectrum of ZnAl-PBSA-LDH revealed a novel peak at 1183 cm^−1^. This peak corresponded to the S−O bond vibration from PBSA, indicating the successful intercalation of PBSA into the LDH layers.

Thermogravimetric analysis (TGA) can provide effective information for the intercalation of PBAS into LDH layers. As shown in Figure S2, the TG-DTG curve of PBSA displayed two weight-loss stages due to the thermal breakdown of water molecules and PBSA. In Figure 2c, ZnAl-NO_3_^−^-LDH showed two main weight-loss stages, corresponding to the thermal decomposition of water molecules and nitrates, respectively [35]. As shown in Figure 2d, ZnAl-PBSA-LDH exhibited four main weight-loss stages. The initial phase of weight loss was attributed to the loss of water molecules and a small amount of nitrate. The second weight loss stage of ZnAl-PBSA-LDH from 200 to 287 °C was associated with the loss of nitrates. The last two weight-loss stages were attributed to the thermal decomposition of PBSA. These results demonstrated that PBSA was successfully inserted into the interlayer of LDHs.

### 2.2. Characterization of Nanocomposite Coatings

**Figure 3:**
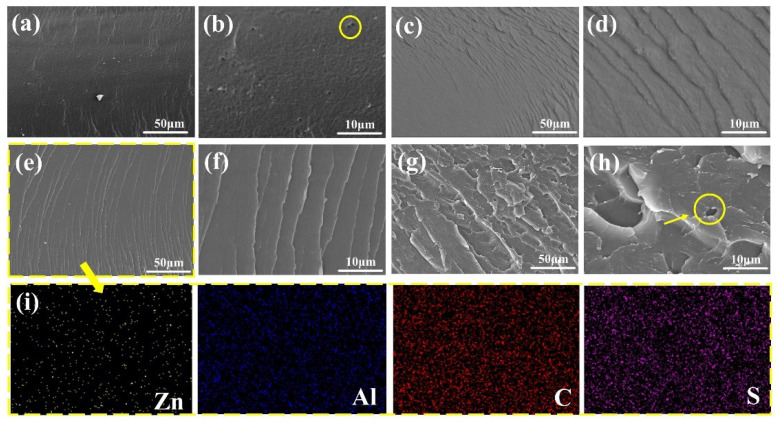
SEM images of the fractured surface of (**a**,**b**) blank WEP, (**c**,**d**) 4-ZPL/WEP, (**e**,**f**) 6-ZPL/WEP, and (**g**,**h**) 8-ZPL/WEP. The EDS mapping of (**i**) 6-ZPL/WEP.

In order to evaluate the compatibility of fillers with coatings, SEM and EDS mappings were used to observe the fracture surface of coatings. For the blank coating, deep holes were present on the smooth fracture surface (yellow circle in Figure 3b). The corrosive ions could easily corrode the steel through these pores. In contrast, the 6-ZPL/WEP coating without holes exhibited homogeneous cracks (Figure 3e), demonstrating the excellent compatibility of ZnAl-PBSA-LDH and WEP coatings, which is beneficial for the improvement of the barrier properties of coatings. However, some agglomerations of ZnAl-PBSA-LDH were observed in the fracture surface of the 8-ZPL/WEP coating (yellow circle in Figure 3h), suggesting the poor dispersion of excessive ZnAl-PBSA-LDH within the WEP coating. Additionally, EDS mapping of the 6-ZPL/WEP coating (Figure 3i) indicated that 0.6 wt.% ZnAl-PBSA-LDH was uniformly distributed across the WEP matrix. The excellent compatibility and dispersion of ZnAl-PBSA-LDH in the WEP coating are conducive to reducing the porosity of the WEP coating, resulting in the improvement of anti-corrosion properties.

### 2.3. Electrochemical Measurements

**Figure 4:**
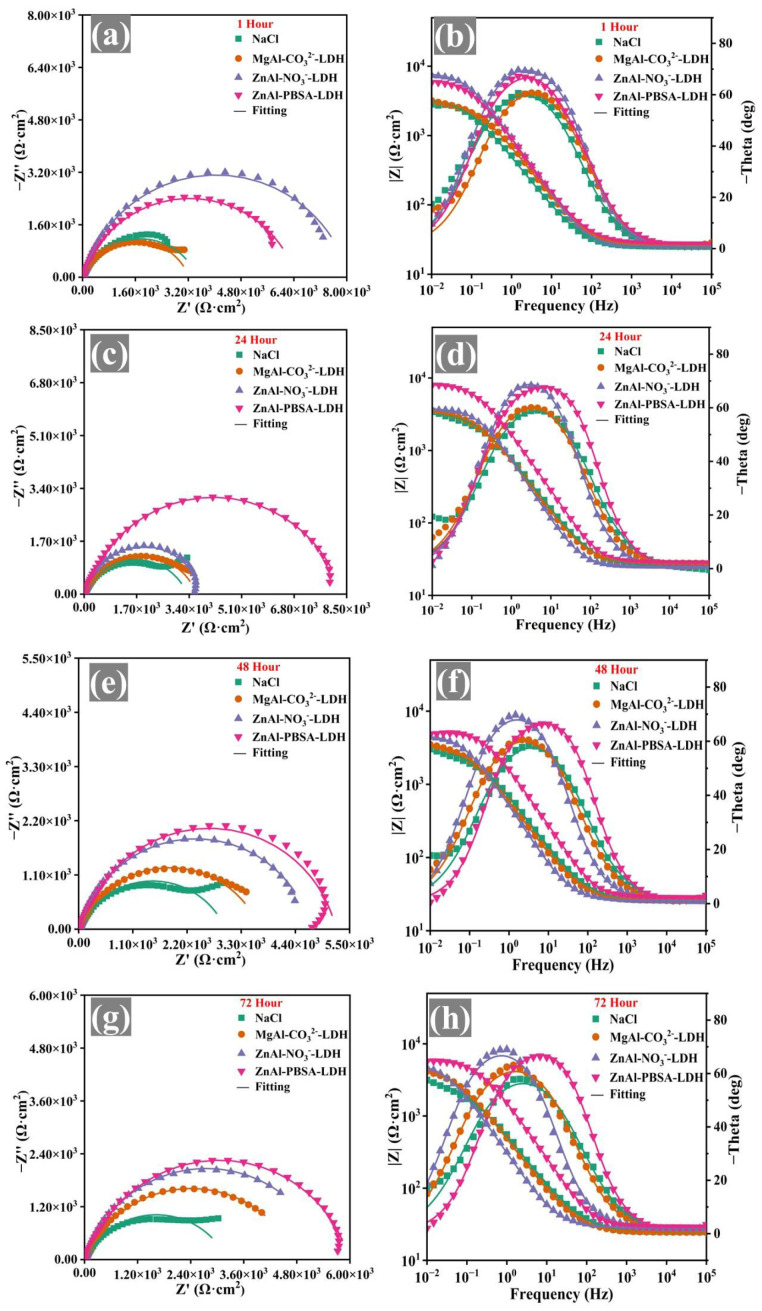
Nyquist plots and Bode-phase plots of the immersed bare steel samples in the blank solution and the extracted solutions of NaCl, MgAl-CO_3_^2−^-LDH, ZnAl-NO_3_^−^-LDH, and ZnAl-PBSA-LDH for (**a**,**b**) 1 h, (**c**,**d**) 24 h, (**e**,**f**) 48 h, and (**g**,**h**) 72 h.

EIS of the solution phase was utilized to explore the corrosion inhibition of LDHs and further select inhibitors that could subsequently be employed to enhance the corrosion resistance of WEP coatings. The corrosion inhibition of the inhibitors was evaluated using low-frequency impedance modulus (|Z_0.01 Hz_|) [36]. During the initial stages of immersion (1 h), the ZnAl-NO_3_^−^-LDH sample exhibited the highest |Z_0.01 Hz_| value among all samples (Figure 4b). However, after 72 h, the |Z_0.01 Hz_| value of ZnAl-NO_3_^−^-LDH significantly decreased (Figure 4h), indicating its limited corrosion inhibition capacity. For MgAl-CO_3_^2−^-LDH, the |Z_0.01 Hz_| value was lower than that of ZnAl-NO_3_^−^-LDH during the corrosion process, implying that MgAl-CO_3_^2−^-LDH exhibited worse corrosion inhibition for Q215 steel. In contrast, the ZnAl-PBSA-LDH sample exhibited the highest impedance modulus among all the samples after 72 h (Figure 4h), demonstrating the excellent corrosion inhibition ability of ZnAl-PBSA-LDH.

The EIS results were fitted with an equivalent circuit by ZSimpwin software and are shown in Table S1 [37]. R_s_ represents the solution resistance. CPE_f_ and R_f_ correspond to film capacitance and resistance. Additionally, CPE_dl_ (double-layer capacitance) and R_ct_ (charge transfer resistance) reflect the underlying interfacial mechanisms. The CPE_dl_ values of steel immersed in ZnAl-PBSA-LDH extract were lower than that of other samples, implying that ZnAl-PBSA-LDH exhibited good corrosion inhibition [38]. To further explore the corrosion inhibition of corrosion inhibitors, the capacitance corresponding to the film and double-layer capacitors was calculated using Equations (1) and (2) [39]. The results are listed in Table S1.

(1) $$C_{f}=Y_{0,f}^{\frac{1}{n}}\times R_{f}^{\frac{\left(1-n\right)}{n}}$$

(2) $$C_{\mathrm{dl}}=Y_{0,\mathrm{dl}}^{\frac{1}{n}}\times {\left(\frac{R_{s}\times R_{\mathrm{ct}}}{R_{s}+R_{\mathrm{ct}}}\right)}^{\frac{\left(1-n\right)}{n}}$$

The C_f_ values were applied to evaluate the barrier performance of a protective film composed of inhibitors and iron ions [36]. During the initial stage of steel immersion in the extracted solution of ZnAl-PBSA-LDH (Table S1), the C_f_ value of the ZnAl-PBSA-LDH sample was found to be the highest compared to the other periods (24 h, 48 h, and 72 h), indicating insufficient barrier performance of the film composed of PBSA and Fe ions. Moreover, the C_f_ values showed a decreasing trend as the immersion time increased, illustrating that the film progressively became denser and thicker. It is because PBSA with heteroatoms (N, S) combined with metal ions and formed complexes [40]. Additionally, the samples with lower C_dl_ values display a higher inhibition effect [36]. The C_dl_ values of ZnAl-PBSA-LDH were the lowest compared to other samples, demonstrating the excellent activity inhibition of ZnAl-PBSA-LDH. Therefore, ZnAl-PBSA-LDH was utilized to improve the anti-corrosion properties of the WEP coating.

**Figure 5:**
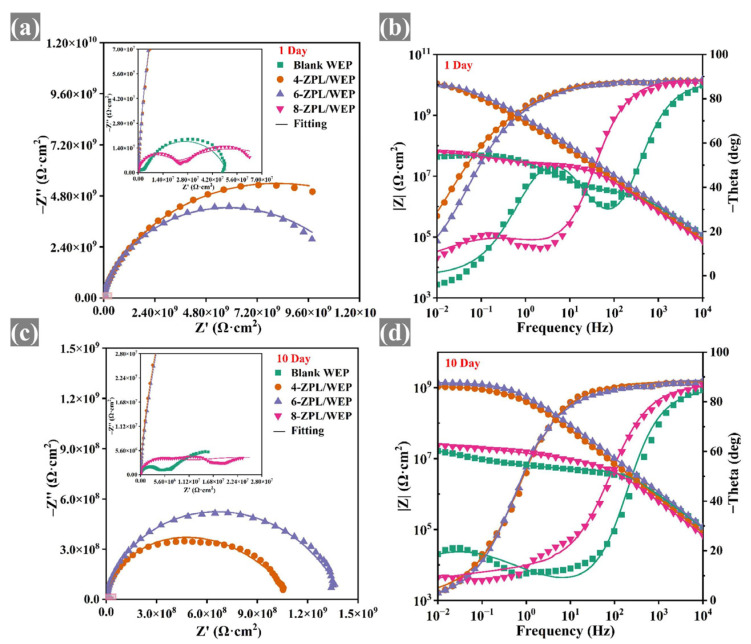
(**a**,**c**) Nyquist and (**b**,**d**) Bode plots of coated samples after 10 days immersion.

**Figure 6:**
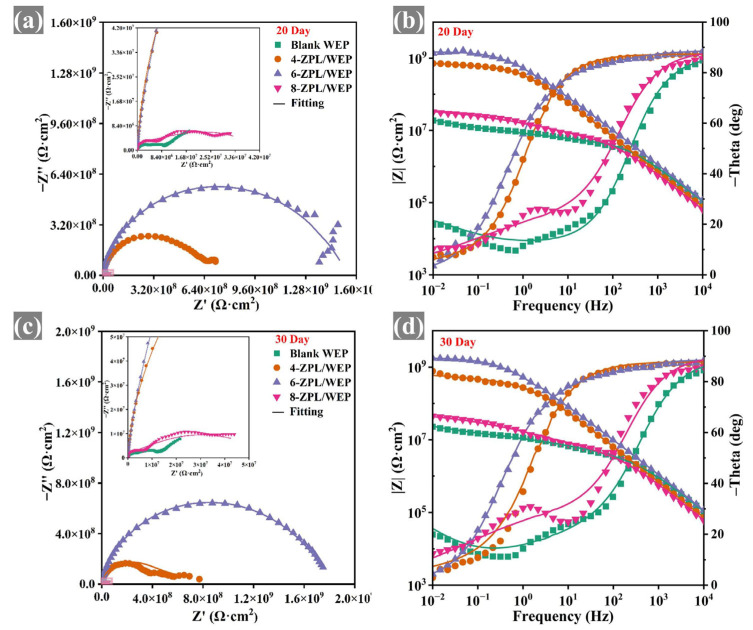
(**a**,**c**) Nyquist and (**b**,**d**) Bode plots of coated samples after 30 days immersion.

EIS testing was conducted to assess the corrosion resistance of coated samples. The large diameter of the capacitor arc in the Nyquist plots indicates that the coatings provided good corrosion protection [41]. As shown in Figure 5, the capacitance arc diameter of composite coatings containing the ZnAl-PBSA-LDH was larger than that of blank coating, indicating that ZnAl-PBSA-LDH could effectively improve the corrosion resistance of the WEP coating. In addition, at a low frequency, the resistive component dominates the impedance of a coating. Hence, the |Z_0.01 Hz_| value is utilized to evaluate the barrier properties of the coatings [42]. In Figure 6, the composite coatings containing ZnAl-PBSA-LDH exhibited a higher |Z_0.01 Hz_| than that of a blank WEP coating after 30 days of immersion, demonstrating that ZnAl-PBSA-LDH can effectively improve the barrier performance of the coating. However, the 8-ZPL/WEP coating exhibited a lower |Z_0.01 Hz_| than that of 0.6 ZnAl-PBSA/WEP coating, which is caused by the aggregation of ZnAl-PBSA-LDH in the WEP coating. The breakpoint frequency (f_b_) was utilized to assess the layered area of the WEP coating and steel [43]. As shown in Figure S3c, the f_b_ value of blank WEP coating increased to 204.6 Hz after soaking for 30 days, implying that blank coating has difficulty effectively protecting the substrate. In contrast, 6-ZPL/WEP coating exhibited a low f_b_ value (0.45 Hz), indicating that ZnAl-PBSA-LDH significantly enhanced the corrosion resistance of coatings. On the one hand, ZnAl-PBSA-LDH can inhibit the diffusion of corrosive ions via the shielding effect; on the other hand, the released PBSA can form a complex film with iron ions that protect the steel.

The equivalent electrical circuit (EEC) models shown in Figure S3d were employed to analyze the EIS results. The parameters R_f_ and R_ct_ represent the resistance of the coating and the charge transfer resistance, respectively. In addition, CPE_c_ and CPE_dl_ correspond to the non-constant phase capacitance and double-layer capacitance of the coating, respectively. R_f_ was applied to evaluate the porosity of the coatings [44]. Figure S3a and Table S2 demonstrate that the blank WEP coating exhibited a low R_f_ value (1.5 × 10^6^ Ω·cm^2^) after 30 days, indicating that the blank WEP coating was ineffective in providing sufficient protection to the substrate. In contrast, the 6-ZPL/WEP coating exhibited a high R_f_ value of 2.52 × 10^7^ Ω·cm^2^, which was 16.8 times that of the blank WEP coating. This suggests that ZnAl-PBSA-LDH can effectively reduce the porosity of the coating and enhance its corrosion resistance. Nevertheless, the R_f_ value of the 8-ZPL/WEP coating (1.9 × 10^6^ Ω·cm^2^) was lower than that of the 6-ZPL/WEP coating after 30 days of immersion, which was attributed to the agglomeration of excessive ZnAl-PBSA-LDH within the WEP coating. In summary, the 6-ZPL/WEP coating had the highest R_f_ and largest capacitance arc diameter among all coatings after 30 days of immersion, illustrating its excellent corrosion resistance.

**Figure 7:**
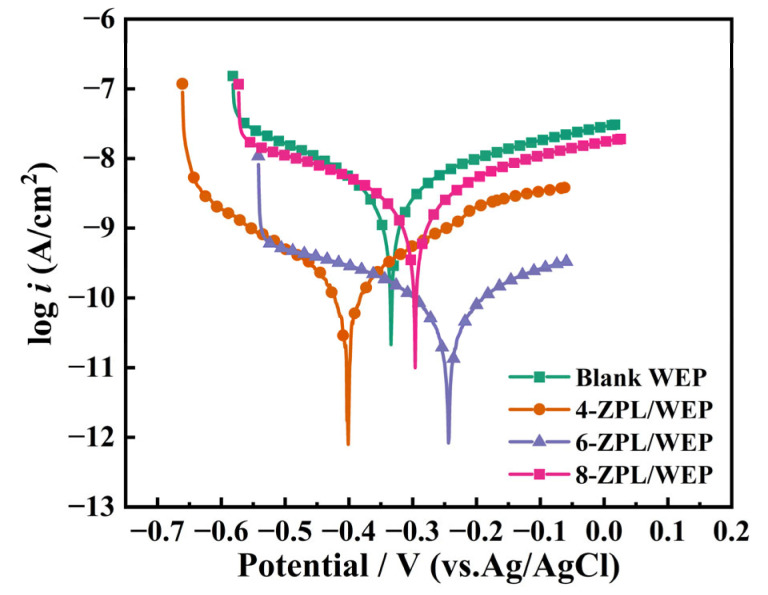
Potentiodynamic polarization curves of all coatings after 30 days of immersion in 3.5 wt.% NaCl solution.

After subjecting the coated samples to a 30-day of immersion, potentiodynamic polarization (PD) testing was employed to further explore the corrosion resistance of the coating. The related results are shown in Figure 7. Subsequently, the corresponding electrochemical parameters were calculated and listed in Table 1, such as corrosion rate (CR), polarization resistance (R_p_), current density (I_corr_), corrosion potential (E_corr_), anode (b_a_), and cathode (b_c_). E_corr_ was utilized to evaluate the possibility of corrosion. In Figure 7, the 6-ZPL/WEP coating exhibited the most positive E_corr_ among all the coatings, implying a lower probability of corrosion for steel coated with the 6-ZPL/WEP coating. In addition, I_corr_ is an important index to use to measure the corrosion resistance of coatings. Generally, a lower I_corr_ value indicates better corrosion protection provided by the coating [44]. As shown in Table 1, the 6-ZPL/WEP coating exhibited the lowest I_corr_ value among all the samples, suggesting excellent corrosion performance. The excellent corrosion resistance of the 6-ZPL/WEP coating can be attributed to multiple contributing factors. Firstly, the ZnAl-PBSA-LDH nanomaterials can effectively prevent the diffusion of the corrosion medium via the shielding effect [45]. Secondly, the favorable compatibility between ZnAl-PBSA-LDH and WEP is beneficial for the construction of the “maze effect”, thereby prolonging the diffusion path of corrosive ions [34]. Thirdly, the released PBSA can be complexed with metal ions to establish a film that can inhibit corrosion; Lastly, LDHs with unique layered structures could attract chloride ions and prevent Cl^−^ from interacting with the metal surface, causing corrosion inhibition [46].

**Figure 8:**
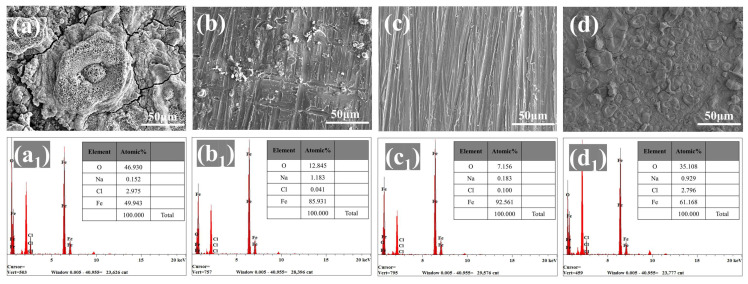
SEM images and EDS mapping of substrates coated by (**a**,**a_1_**) blank WEP, (**b**,**b_1_**) 4-ZPL/WEP, (**c**,**c_1_**) 6-ZPL/WEP, and (**d**,**d_1_**) 8-ZPL/WEP coatings after immersion of 30 days.

SEM and EDS were applied to evaluate the degree of corrosion of the substrate after 30 days of immersion. Figure 8a depicts the substantial amount of rust that appeared on the surface of the steel protected by the blank WEP coating, illustrating the poor corrosion protection provided by the blank WEP coating. In contrast, a small loose rust layer appeared on the surface of the substrate coated with the 4-ZPL/WEP coating (Figure 8b), implying that ZnAl-PBSA-LDH with its shielding effect and activity inhibition can effectively improve the anti-corrosion properties of WEP. For the steel coated with the 6-ZPL/WEP coating, there were no noticeable signs of corrosion, indicating that the 6-ZPL/WEP coating exhibited excellent protection. Furthermore, EDS was utilized to measure the oxygen content of the substrate surface to assess the degree of corrosion [47]. Figure 8(c_1_) showed that the surface of the steel coated with the 6-ZPL/WEP coating exhibited a low oxygen content (7.1%), which was decreased by 84.6% compared to that of the blank WEP coating (46%), indicating that the 6-ZPL/WEP coating provides excellent protection for the substrate. Chloride ions play a major role in the corrosion process as they can adsorb onto the metal surface, causing damage to the metal surface [48]. As shown in Figure 8, the chloride ion content of the composite coatings was lower than that of the blank WEP coating, which is caused by the unique layered structure of LDHs. LDHs can selectively adsorb and immobilize chloride ions via electrostatic interactions, further hindering the diffusion of chloride ions and protecting the steel.

**Figure 9:**
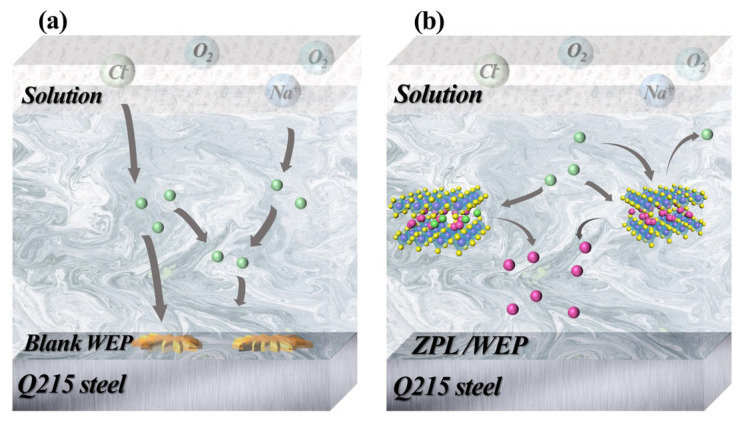
Anti-corrosion mechanism of (**a**) blank WEP and (**b**) ZPL/WEP.

As shown in Figure 9a, corrosive media such as H_2_O and Cl^−^ can easily spread to steel surfaces from the micropores and microcracks of the blank WEP coating, resulting in corrosion. In contrast, ZnAl-PBSA-LDH nanomaterials may effectively stop the spread of the corrosion medium through the shielding effect. Simultaneously, the released PBSA could combine with the metal ion and adsorb on the surface of the substrate, resulting in a complex film that protects the substrate from corrosion. Additionally, ZnAl-PBSA-LDH captures Cl^−^ in solution, thereby reducing the interaction between Cl^−^ and steel [24]. In summary, ZnAl-PBSA-LDH, with its shielding effects and activity inhibition, can effectively improve the anticorrosion properties of the coating.

## 3. Materials and Methods

### 3.1. Materials

Zinc chloride (ZnCl_2_), aluminum chloride hexahydrate (AlCl_3_·6H_2_O), magnesium chloride hexahydrate (MgCl_2_·6H_2_O), sodium carbonate (Na_2_CO_3_), sodium nitrate (NaNO_3_), and sodium hydroxide (NaOH) were purchased from Macklin Biochemical Co., Ltd. (Shanghai, China) PBSA was purchased from Yuanye Bio-Technology Co., Ltd. (Shanghai, China). The Q215 steel used in the study was purchased from Oudifu Co., Ltd. The elemental content (wt.%) of Q215 steel (abbreviated Q215) was found to be: C ≤ 0.15; S ≤ 0.05; P ≤ 0.05; Si ≤ 0.35; Mn ≤ 1.20; and balance Fe. E51 (epoxide equivalent weight = 196) was obtained from Dongfeng Chemicals Co., Ltd., Guangzhou, China. The curing agent used was BANCO901, which has an active hydrogen equivalent value of 248, supplied by B & C Chemicals Co., Ltd. (Shanghai, China).

### 3.2. Preparation of MgAl-CO_3_^2−^-LDH

The MgAl-CO_3_^2−^-LDH was synthesized using the conventional coprecipitation method. Initially, a mixture of 0.5 M MgCl_2_·6H_2_O and 0.25 M AlCl_3_·6H_2_O (V = 50 mL) was slowly added dropwise into a solution of 0.25 M Na_2_CO_3_ (V = 100 mL) while adjusting the pH to 9 ± 0.5 with sodium hydroxide, then stirring for 2 h at 35 °C. The resulting white precipitate was then crystallized at 70 °C for 24 h. Subsequently, the mixture solution was filtered to remove all supernatants, and the sample was washed several times with distilled water. Finally, the sample was dried in a vacuum oven at 80 °C.

### 3.3. Preparation of ZnAl-NO_3_^−^-LDH

The preparation of *ZnAl-NO_3_^−^-LDH* needs to be completed under a nitrogen atmosphere. Initially, a mixture of 0.5 M ZnCl_2_·6H_2_O and 0.25 M AlCl_3_·6H_2_O (V = 50 mL) was slowly added dropwise into a solution of 0.25 M NaNO_3_ (V = 100 mL) while adjusting the pH to 9 ± 0.5 with sodium hydroxide and stirring for 2 h at 35 °C. Then, the resulting mixture solution was left at 70 °C for 24 h. Subsequently, the mixture solution was filtered to remove all supernatants, and the sample was washed several times with distilled water. Finally, the sample was dried in a vacuum oven at 80 °C to obtain ZnAl-NO_3_^−^-LDH.

### 3.4. Preparation of ZnAl-LDHs Loaded with PBSA

The preparation of *ZnAl-PBSA-LDH* needs to be completed under a nitrogen atmosphere. First, the determined amounts of ZnCl_2_ solution and AlCl_3_·6H_2_O solution were added slowly to the flask via a constant pressure drip funnel under vigorous stirring at a temperature of 35 °C. Subsequently, 100 mL of PBSA solution was slowly dropped into the mixed solution. Then, sodium hydroxide was used to adjust the pH of the solution to 9. The mixed solution was stirred for 2 h, following which the white precipitate was subjected to aging at a temperature of 70 °C for 24 h. Subsequently, the mixture was filtered to remove all the supernatant. The resulting sample was washed several times using degassed distilled water and then dried at 80 °C in a vacuum oven.

### 3.5. Coating Preparation

**Figure 10:**
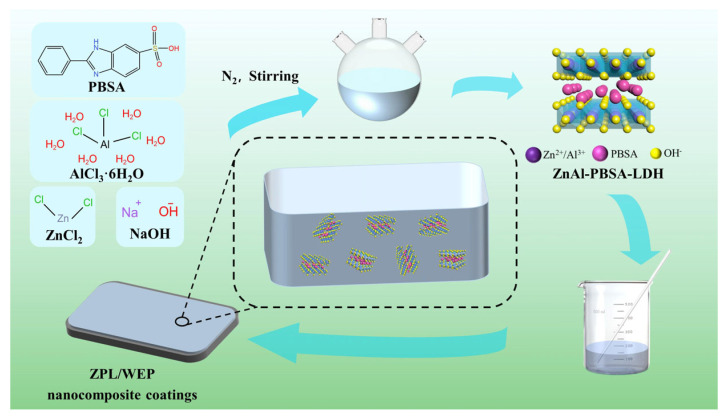
Schematic diagram of the synthesis and preparation of the LDH coating.

The Q215 substrate, measuring 120 mm × 120 mm × 1 mm, underwent a preparation process before the application of the composite coating. Initially, the substrate was polished using sandpaper ranging from 180 to 600 grades. Subsequently, the substrate was degreased using ethanol. The composite coating was made by mixing BANCO901, E51, deionized water, and fillers (ZnAl-PBSA-LDH of 0.4 wt.%, 0.6 wt.%, 0.8 wt.%) using sonication for 10 min. The composite coatings were applied to the pre-treated Q215 substrate by a four-sided applicator with a wet thickness of 100 μm. After drying at room temperature for 7 days, the coating was further dried at 75 °C for 6 h. The final thickness of the dried coating was 40 ± 2 μm. The experimental flow chart is shown in Figure 10.

### 3.6. Characterization

Fourier transform infrared (FTIR) analysis was performed using the Thermo Fisher Scientific Nicolet iS50R Spectrometer (Waltham, MA, USA). The phase composition was analyzed by X-ray diffraction (XRD) using the Rigaku MiniFlex600, which uses Cu-Kα radiation (λ = 0.15406 nm) in a scanning range of 2θ = 3° to 70° at a rate of 10°/min. Furthermore, the thermal stability of the samples was investigated by thermogravimetric (TG) using the Netzsch STA449F5 Jupiter, in the N2 atmosphere with a temperature range of 25–800 °C and a heating rate of 10 °C/min. The microstructure and morphology of fillers were characterized by field emission scanning electron microscopy (FESEM, Apreo 2S HiVac, Waltham, MA, USA). Moreover, the fracture surface of the coatings was studied using energy dispersive spectroscopy (EDS, Bruker XFlash, Billerica, MA, USA) in combination with SEM (JEOL S-3400N, Tokyo, Japan).

### 3.7. Electrochemical Measures

The electrochemical impedance spectroscopy (EIS) test was conducted to evaluate the effect of the extract on bare metal corrosion in NaCl (3.5 wt.%) solution. The concentration of the inhibitors in the extracted solutions is all 0.4 wt.%. Furthermore, EIS and potentiodynamic polarization (PD) tests were utilized to evaluate the anticorrosion properties of the coating in 3.5 wt.% NaCl. The EIS and PD tests were performed using the CHI760E electrochemical workstation and a device consisting of Ag/AgCl, graphite, and steel with an exposed area of 3.14 cm^2^. The EIS and PD tests were conducted using a stable open-circuit potential and an amplitude of 10 mV across a frequency range of 10^5^–10^−2^ Hz. After 30 days of immersion, the corrosion products of the intact coated samples were observed using SEM.

## 4. Conclusions

This study successfully synthesized ZnAl-PBSA-LDH materials using a simple one-step approach. The successful preparation of ZnAl-PBSA-LDH was confirmed by SEM, XRD, and FTIR. Then, different amounts of ZnAl-PBSA-LDH were incorporated into the WEP coating to prepare a high-performance anti-corrosion coating. The SEM and EDS results showed that ZnAl-PBSA-LDH exhibited good dispersibility with the WEP coating. The results of electrochemical measures showed that the 6-ZPL/WEP coating exhibited a high |Z_0.01 Hz_| value (10^9.24^ Ω·cm^2^), which was almost two orders of magnitude higher than that of the blank WEP coating. This indicates that ZnAl-PBSA-LDH could effectively improve the corrosion resistance of the coating due to its shielding effect, activity inhibition, and ion exchange. Overall, we have successfully used this feature to produce lightweight, high-performance WEP composite coatings, highlighting the significant potential of LDHs in the field of anti-corrosion.

## Figures and Tables

**Table 1:** Tafel extrapolation results obtained from Figure 7.

Sample	*E_corr_* (mV)	*I_corr_* (A/cm^2^)	R_p_ (Ω·cm^2^)	b_a_ (mV/dec)	b_c_ (mV/dec)	CR (mpy)
Blank WEP	−3340	3.225 × 10^−9^	1.29 × 10^7^	209.12	−176.55	4.72 × 10^−4^
4-ZPL/WEP	−4010	1.986 × 10^−10^	1.93 × 10^8^	175.60	−177.56	2.90 × 10^−5^
6-ZPL/WEP	−2440	8.012 × 10^−11^	5.49 × 10^8^	205.55	−198.93	1.17 × 10^−5^
8-ZPL/WEP	−2960	2.286 × 10^−9^	1.91 × 10^7^	199.40	−203.42	3.34 × 10^−4^

## Data Availability

Data of the present study are available in the article.

## Supplementary Materials

The following supporting information can be downloaded at: https://www.mdpi.com/article/10.3390/molecules28135199/s1, Figure S1: SEM images of ZnAl-NO_3_^−^-LDH. Figure S2: TG-DTG curves of (a) MgAl-CO_3_^2−^-LDH and (b) PBSA. Figure S3: The changes of (a) Rf values, (b) |Z_0.01 Hz_| values, (c) fb values; (d) the equivalent electrical circuits. Table S1: The parameters obtained from the fitting of EIS data are related to the uncoated samples exposed to the blank and inhibited solutions. Table S2: The parameters obtained from the fitting of EIS data related to the coated samples immersed in 3.5 wt.% NaCl solution.

## Sample Availability

Samples of the compounds are available from the authors.
