# Supplementary material for: A Waterborne Epoxy Composite Coating with Smart Corrosion Resistance Based on 2-Phenylbenzimidazole-5-sulfonic Acid/Layered Double Hydroxide Composite
Source: Molecules. 2023 Jul 4;28(13):5199. doi: 10.3390/molecules28135199 (PMC10343620; doi:10.3390/molecules28135199)
Supplement: Supplementary file 1 [file molecules-28-05199-s001.zip › molecules-2433665-supplementary.pdf]

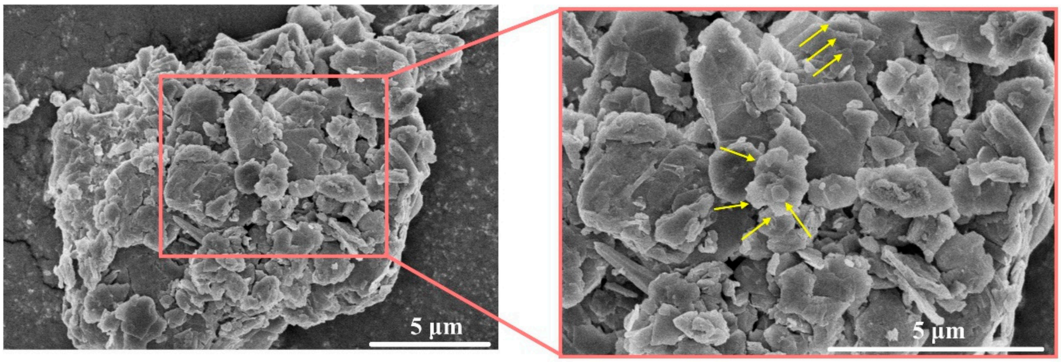

Figure S1 SEM images of ZnAl-NO_3^--LDH

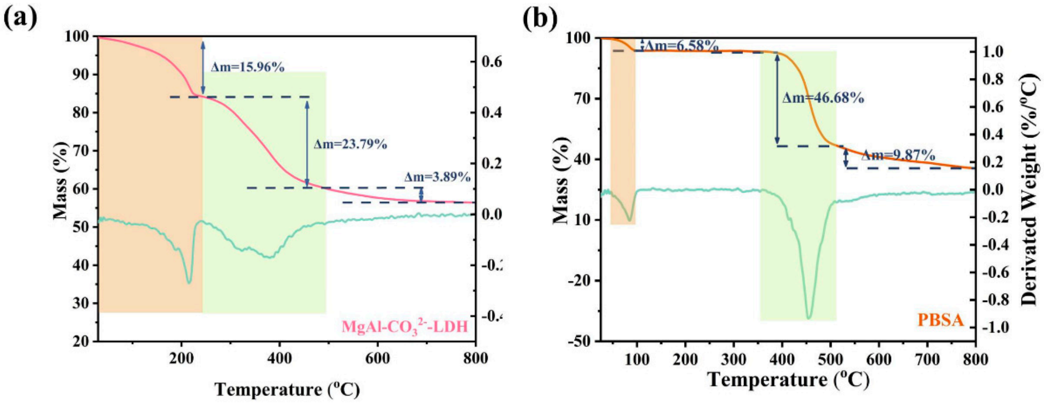

Figure S2 TG-DTG curves of (**a**) MgAl-CO_3^{2-}-LDH and (b) PBSA.

Table S1. The parameters obtained from the fitting of EIS data are related to the uncoated samples exposed to the blank and inhibited solutions.

Specimens	Rs ($\Omega\cdot cm^2$)	CPE_c		R_f ($\Omega\cdot cm^2$)	C_f ($F\cdot cm^{-2}$)	CPE_{dl}		R_{ct} ($\Omega\cdot cm^2$)	C_{dl} ($F\cdot cm^{-2}$)	log|Z| ($\Omega\cdot cm^2$)	Chi squared
		Y_c ($F\cdot cm^{-2}\cdot s^{n-1}$)	n			Y_{dl} ($F\cdot cm^{-2}\cdot s^{n-1}$)	n				
**NaCl-1H**	**24.51**	**-**	**-**	**-**	**-**	**4.91×10^{-4}**	**0.76**	**3453**	**1.20×10^{-4}**	**3.45**	**3.01×10^{-3}**
**NaCl-24H**	**25.09**	**-**	**-**	**-**	**-**	**3.02×10^{-4}**	**0.75**	**3314**	**5.97×10^{-5}**	**3.55**	**4.51×10^{-3}**
**NaCl-48H**	**25.39**	**-**	**-**	**-**	**-**	**3.51×10^{-4}**	**0.74**	**2958**	**6.86×10^{-5}**	**3.49**	**3.32×10^{-3}**
**NaCl-72H**	**24.32**	**-**	**-**	**-**	**-**	**4.82×10^{-4}**	**0.72**	**3203**	**8.60×10^{-5}**	**3.50**	**2.04×10^{-3}**
**MgAl-CO_3^{2-}-1H**	**25.70**	**-**	**-**	**-**	**-**	**3.34×10^{-4}**	**0.77**	**3201**	**8.13×10^{-5}**	**3.50**	**2.06×10^{-3}**
**MgAl-CO_3^{2-}-24H**	**27.53**	**-**	**-**	**-**	**-**	**3.17×10^{-4}**	**0.77**	**3617**	**7.48×10^{-5}**	**3.55**	**1.15×10^{-3}**

**MgAl-CO_3^{2-}-48H**	**25.81**	**-**	**-**	**-**	**-**	**4.05×10^{-4}**	**0.76**	**3658**	**9.69×10^{-5}**	**3.54**	**6.59×10^{-4}**
**MgAl-CO_3^{2-}-72H**	**24.63**	**-**	**-**	**-**	**-**	**5.05×10^{-4}**	**0.76**	**4772**	**1.24×10^{-4}**	**3.62**	**5.59×10^{-4}**
**ZnAl-NO_3^--1H**	**25.15**	**-**	**-**	**-**	**-**	**2.27×10^{-4}**	**0.84**	**8048**	**8.43×10^{-5}**	**3.87**	**3.12×10^{-4}**
**ZnAl-NO_3^--24H**	**24.95**	**-**	**-**	**-**	**-**	**2.63×10^{-4}**	**0.86**	**3789**	**1.18×10^{-4}**	**3.55**	**4.64×10^{-4}**
**ZnAl-NO_3^--48H**	**26.00**	**-**	**-**	**-**	**-**	**4.08×10^{-4}**	**0.84**	**4697**	**1.76×10^{-4}**	**3.65**	**5.70×10^{-4}**
**ZnAl-NO_3^--72H**	**28.18**	**-**	**-**	**-**	**-**	**7.19×10^{-4}**	**0.83**	**5454**	**3.17×10^{-4}**	**3.67**	**1.51×10^{-3}**
**ZnAl-PBSA-1H**	**27.35**	**2.28×10^{-4}**	**0.81**	**5.33×10^{2}**	**1.38×10^{-4}**	**1.28×10^{-5}**	**0.98**	**5915**	**1.13×10^{-5}**	**3.77**	**4.05×10^{-4}**
**ZnAl-PBSA-24H**	**28.16**	**8.78×10^{-5}**	**0.88**	**1.47×10^{3}**	**6.66×10^{-5}**	**5.79×10^{-5}**	**0.70**	**6870**	**3.84×10^{-6}**	**3.90**	**2.44×10^{-4}**
**ZnAl-PBSA-48H**	**28.15**	**9.41×10^{-5}**	**0.87**	**1.70×10^{3}**	**7.20×10^{-5}**	**5.51×10^{-5}**	**0.81**	**3500**	**1.26×10^{-5}**	**3.68**	**6.12×10^{-4}**
**ZnAl-PBSA-72H**	**28.81**	**1.11×10^{-4}**	**0.86**	**2.95×10^{3}**	**9.17×10^{-5}**	**9.61×10^{-5}**	**0.77**	**3000**	**1.57×10^{-5}**	**3.76**	**3.24×10^{-4}**

Table S2. The parameters obtained from the fitting of EIS data related to the coated samples immersed in 3.5 wt.% NaCl solution.

**Specimens**	**CPE_c**		**R_f**	**CPE_{dl}**		**R_{ct}**	**Log |Z|**	**Chi squared**
	**Y_c ($F\cdot cm^{-2}\cdot s^{n-1}$)**	**n**	**($\Omega\cdot cm^2$)**	**Y_{dl} ($F\cdot cm^{-2}\cdot s^{n-1}$)**	**n**	**($\Omega\cdot cm^2$)**	**($\Omega\cdot cm^2$)**	
**Blank WEP-1day**	**1.83×10^{-10}**	**0.98**	**3.26×10^{6}**	**5.93×10^{-9}**	**0.81**	**4.69×10^{7}**	**7.63**	**3.86×10^{-3}**
**Blank WEP-10day**	**2.62×10^{-10}**	**0.95**	**4.57×10^{6}**	**1.90×10^{-7}**	**0.45**	**3.06×10^{7}**	**7.22**	**6.05×10^{-3}**
**Blank WEP-20day**	**2.19×10^{-10}**	**0.96**	**2.00×10^{6}**	**7.15×10^{-8}**	**0.24**	**2.00×10^{7}**	**7.28**	**3.56×10^{-3}**
**Blank WEP-30day**	**1.65×10^{-10}**	**0.99**	**1.50×10^{6}**	**2.56×10^{-8}**	**0.38**	**1.60×10^{7}**	**7.36**	**2.56×10^{-3}**
**4-ZPL/WEP-1day**	**2.38×10^{-10}**	**0.98**	**8.32×10^{8}**	**2.02×10^{-10}**	**0.58**	**1.68×10^{10}**	**10.04**	**1.28×10^{-3}**
**4-ZPL/WEP-10day**	**2.41×10^{-10}**	**0.98**	**1.50×10^{7}**	**3.70×10^{-10}**	**0.45**	**1.16×10^{9}**	**9.02**	**1.41×10^{-3}**
**4-ZPL/WEP-20day**	**2.89×10^{-10}**	**0.97**	**1.30×10^{7}**	**7.46×10^{-10}**	**0.21**	**1.00×10^{9}**	**8.85**	**1.31×10^{-3}**
**4-ZPL/WEP-30day**	**2.82×10^{-10}**	**0.98**	**1.25×10^{7}**	**1.07×10^{-9}**	**0.27**	**8.00×10^{8}**	**8.89**	**5.48×10^{-3}**

6-ZPL/WEP-1day	1.66×10^{-10}	0.98	8.00×10^{8}	1.36×10^{-10}	0.58	1.20×10^{10}	10.01	1.19×10^{-3}
6-ZPL/WEP-10day	1.58×10^{-10}	0.99	2.71×10^{7}	2.10×10^{-10}	0.61	1.40×10^{9}	9.13	9.97×10^{-4}
6-ZPL/WEP-20day	1.61×10^{-10}	0.99	2.60×10^{7}	2.30×10^{-10}	0.62	1.53×10^{9}	9.14	1.43×10^{-3}
6-ZPL/WEP-30day	1.63×10^{-10}	0.99	2.52×10^{7}	2.48×10^{-10}	0.63	1.80×10^{9}	9.24	1.07×10^{-3}
8-ZPL/WEP-1day	2.55×10^{-10}	0.99	1.82×10^{7}	3.06×10^{-8}	0.47	6.86×10^{7}	7.80	2.93×10^{-3}
8-ZPL/WEP-10day	3.33×10^{-10}	0.97	2.80×10^{6}	4.09×10^{-8}	0.24	4.20×10^{7}	7.38	4.00×10^{-3}
8-ZPL/WEP-20day	2.96×10^{-10}	0.98	2.70×10^{6}	2.67×10^{-8}	0.37	4.10×10^{7}	7.51	4.24×10^{-3}
8-ZPL/WEP-30day	2.61×10^{-10}	1.00	1.90×10^{6}	2.57×10^{-8}	0.38	6.50×10^{7}	7.65	9.38×10^{-3}

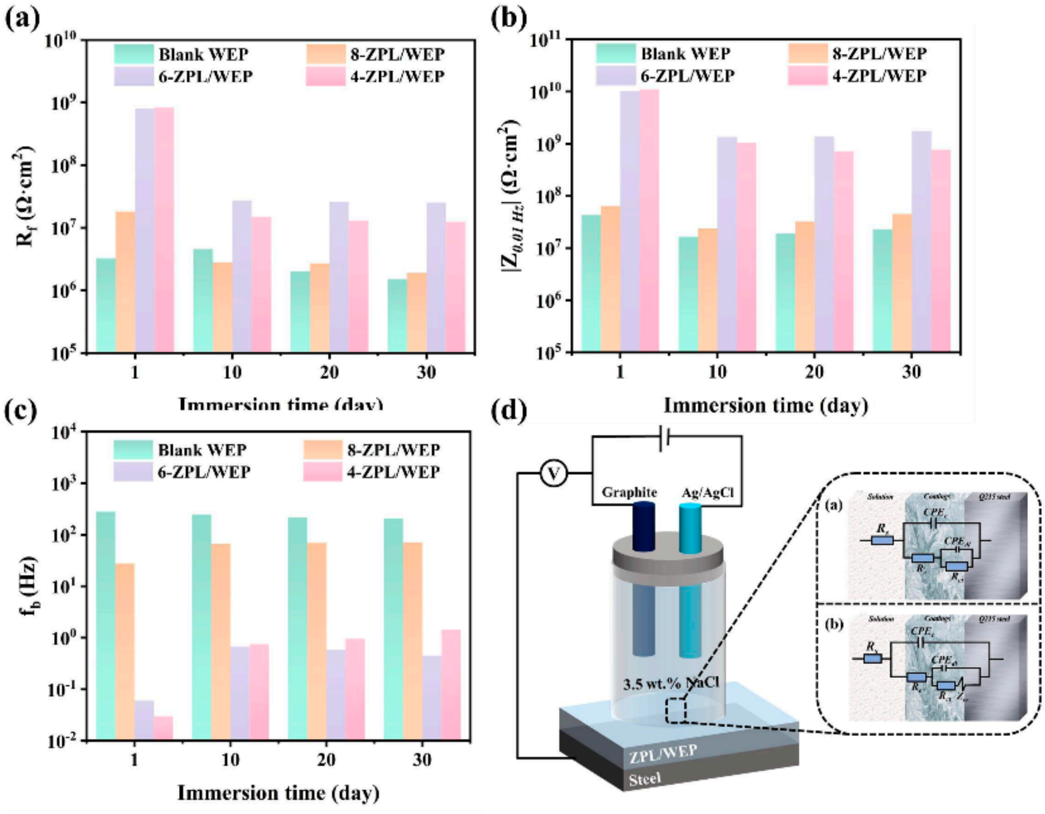

Figure S3. The changes of (**a**) R_f values, (**b**) $|Z_{0.01\ Hz}|$ values, (**c**) f_b values; (**d**) the equivalent electrical circuits.
